# Outcome Definition Influences the Relationship between Genetic Polymorphisms of *ERCC1*, *ERCC2*, *SLC22A2* and Cisplatin Nephrotoxicity in Adult Testicular Cancer Patients

**DOI:** 10.3390/genes10050364

**Published:** 2019-05-10

**Authors:** Zulfan Zazuli, Leila S. Otten, Britt I. Drögemöller, Mara Medeiros, Jose G. Monzon, Galen E. B. Wright, Christian K. Kollmannsberger, Philippe L. Bedard, Zhuo Chen, Karen A. Gelmon, Nicole McGoldrick, Abhijat Kitchlu, Susanne J. H. Vijverberg, Rosalinde Masereeuw, Colin J. D. Ross, Geoffrey Liu, Bruce C. Carleton, Anke H. Maitland-van der Zee

**Affiliations:** 1 Department of Respiratory Medicine, Amsterdam University Medical Centers, University of Amsterdam, 1105 AZ Amsterdam, The Netherlands; z.zazuli@amc.uva.nl (Z.Z.); s.j.vijverberg@amsterdamumc.nl (S.J.H.V.); 2 Department of Pharmacology-Clinical Pharmacy, School of Pharmacy, Bandung Institute of Technology, Bandung 40132, Indonesia; 3 Division of Pharmacology, Utrecht Institute for Pharmaceutical Sciences, Utrecht University, 3512 JE Utrecht, The Netherlands; l.s.otten@students.uu.nl (L.S.O.); R.Masereeuw@uu.nl (R.M.); 4 British Columbia Children’s Hospital Research Institute, Vancouver, BC V5Z 4H4, Canada; bdrogemoller@cmmt.ubc.ca (B.I.D.); gwright@cmmt.ubc.ca (G.E.B.W.); colin.ross@ubc.ca (C.J.D.R.); 5 Faculty of Pharmaceutical Sciences, The University of British Columbia, Vancouver, BC V6T 1Z4, Canada; 6 Nephrology Research Unit, Hospital Infantil de México Federico Gómez, Mexico City 06720, Mexico; medeiro.mara@gmail.com; 7 Departamento de Farmacología, Facultad de Medicina, Universidad Nacional Autónoma de México, Mexico City 04510, Mexico; 8 Department of Medical Oncology, Tom Baker Cancer Centre, Calgary, AB T2N 4N2, Canada; jgmonzon@ucalgary.ca; 9 Department of Medical Genetics, University of British Columbia, Vancouver, BC V6T 1Z4, Canada; 10 BC Cancer Agency and University of British Columbia, Vancouver, BC V6T 1Z4, Canada; ckollmannsberger@bccancer.bc.ca (C.K.K.); kgelmon@bccancer.bc.ca (K.A.G.); 11 Princess Margaret Cancer Centre and University of Toronto, Toronto, ON M5S, Canada; philippe.bedard@uhn.ca; 12 Medical Oncology and Hematology, Department of Medicine, Princess Margaret Cancer Centre-University Health Network and University of Toronto, Toronto, ON M5S, Canada; Zhuo.Chen@uhnresearch.ca (Z.C.); Geoffrey.Liu@uhn.ca (G.L.); 13 Pharmaceutical Outcomes Programme, BC Children’s Hospital, Vancouver, BC V6H 3N1, Canada; nicolemcgoldrick@hotmail.com (N.M.); bcarleton@popi.ubc.ca (B.C.C.); 14 Division of Nephrology, Department of Medicine, University Health Network and University of Toronto, Toronto, ON M5S, Canada; Abhijat.Kitchlu@uhn.ca; 15 Division of Translational Therapeutics, Department of Pediatrics, Faculty of Medicine, University of British Columbia, Vancouver, BC V6T 1Z4, Canada

**Keywords:** pharmacogenetics, cisplatin, nephrotoxicity, kidney injury, genetic polymorphisms

## Abstract

Although previous research identified candidate genetic polymorphisms associated with cisplatin nephrotoxicity, varying outcome definitions potentially contributed to the variability in the effect size and direction of this relationship. We selected genetic variants that have been significantly associated with cisplatin-induced nephrotoxicity in more than one published study (*SLC22A2* rs316019; *ERCC1* rs11615 and rs3212986; *ERCC2* rs1799793 and rs13181) and performed a replication analysis to confirm associations between these genetic polymorphisms and cisplatin nephrotoxicity using various outcome definitions. We included 282 germ cell testicular cancer patients treated with cisplatin from 2009–2014, aged >17 years recruited by the Canadian Pharmacogenomics Network for Drug Safety. Nephrotoxicity was defined using four grading tools: (1) Common Terminology Criteria for Adverse Events (CTCAE) v4.03 for acute kidney injury (AKI) or CTCAE-AKI; (2) adjusted cisplatin-induced AKI; (3) elevation of serum creatinine; and (4) reduction in the estimated glomerular filtration rate (eGFR). Significant associations were only found when using the CTCAE v4.03 definition: genotype CA of the *ERCC1* rs3212986 was associated with decreased risk of cisplatin nephrotoxicity (OR_adj_ = 0.24; 95% CI: 0.08–0.70; *p* = 0.009) compared to genotype CC. In contrast, addition of allele A at *SLC22A2* rs316019 was associated with increased risk (OR_adj_ = 4.41; 95% CI: 1.96–9.88; *p* < 0.001) while genotype AC was associated with a higher risk of cisplatin nephrotoxicity (OR_adj_ = 5.06; 95% CI: 1.69–15.16; *p* = 0.004) compared to genotype CC. Our study showed that different case definitions led to variability in the genetic risk ascertainment of cisplatin nephrotoxicity. Therefore, consensus on a set of clinically relevant outcome definitions that all such studies should follow is needed.

## 1. Introduction

Cisplatin remains one of the most widely prescribed antineoplastic therapies due to its effectiveness as a component of first-line regimens against various types of cancers, including carcinomas, germ cell tumours, lymphomas and sarcomas [1,2]. In Europe, the 1- and 5-years survival rate in testicular cancer patients was 98% and 97%, respectively [3]. However, the dose-limiting toxicities of cisplatin, such as nausea and vomiting, hematotoxicity, ototoxicity and nephrotoxicity, hinder its potential antineoplastic effect. Nephrotoxicity is the most prevalent of these adverse effects caused by cisplatin, resulting in a two-fold risk of acute kidney injury and an increase in serum creatinine levels [4,5]. Approximately one third of all patients treated with cisplatin develop renal dysfunction after a single dosage of cisplatin (50–100 mg/m^2^) [6]. In addition, concerns about long-term renal side effects are rising especially in cancers that occur in young patients and have a high chance of being successfully treated such as testicular cancer [7]. A previous study suggested that circulating platinum is still detectable in the plasma of testicular cancer survivors even 20 years after the last administration of cisplatin [8].

Cisplatin is mainly excreted through the kidneys. Therefore, renal tubular injury is a common clinical manifestation of cisplatin accumulation in renal tubular cells. Cisplatin levels in tubular epithelial cells may increase up to five times higher levels than blood levels [9]. After uptake via organic cation transporter 2 (OCT2) and high-affinity copper transporter 1 (CTR1) in the renal tubules, multiple mechanisms lead to cytotoxicity: complex intracellular pathways lead to DNA damage and cell death and an inflammatory response speeds up renal damage even more [10]. Cisplatin may induce vascular injury as well, which accelerates tubular cell death. These multifactorial processes lead to tubular necrosis and eventually loss of kidney function [10]. This loss of function manifests itself in multiple ways: acute kidney injury (as measured by decreased glomerular filtration rate (GFR)), decreased magnesium and potassium levels and increased serum creatinine (SCr) are paramount but cisplatin may also cause hypocalcaemia, renal salt wasting and even chronic kidney disease [10]. Various patient-related (e.g., age, gender, chronic comorbid illness, pre-existing kidney disease) and treatment-related factors (cisplatin dose per cycle, cumulative dosage, hydration) have been associated with cisplatin nephrotoxicity [11]. In addition, previous studies also suggest that variations in genes involved in cisplatin pharmacodynamics and pharmacokinetics contribute to cisplatin nephrotoxicity [12,13,14,15,16,17,18].

Genetic variations have been reported to play a role both as protective and as risk factors for cisplatin nephrotoxicity. In a recent systematic review we reported that variants in *ERCC1*, *ERCC2* and *SLC22A2* genes were associated with cisplatin nephrotoxicity and replicated in at least one other study [19]. *ERCC1* and *ERCC2* polymorphisms have been associated with alterations of DNA repair process in cells [20,21,22] including possibly the nephron following cisplatin exposure [19]. In addition, *ERCC1* polymorphisms may alter cell sensitivity to cisplatin [23]. Polymorphisms in *SLC22A2,* a gene which product is the organic cation transporter OCT2 responsible for cellular cisplatin uptake in renal proximal tubule cells [24,25], affects the severity of tubular injury process due to cisplatin accumulation. However, variability in effect size and direction of association have been reported. Consequently, this complicates the understanding of the true impact of genetic variants. Differences in clinical characteristics for example, age, type of cancer, cisplatin dose and ethnicity might be related to variability of results. We expect that differences in how cisplatin nephrotoxicity is defined contribute to the variability in results as no widely accepted single cisplatin nephrotoxicity definition exists.

Our aim is to validate the use of already associated genetic variants to predict cisplatin nephrotoxicity and to determine if different cisplatin nephrotoxicity definitions contributed to the variability in effect size and direction of already published associations between these genetic polymorphisms and cisplatin nephrotoxicity. This approach was important to highlight the need of consensus on a set of clinically relevant cisplatin nephrotoxicity definitions that future studies is able to follow.

## 2. Materials and Methods

This study is reported according to Strengthening the Reporting of Genetic Association Studies (STREGA) guidelines [26].

### 2.1. Study Design and Participants

The retrospective study included males (≥17 years old) diagnosed with germ cell testicular cancer treated with cisplatin between January 1979 and February 2013. These patients were part of a previously conducted study on cisplatin-induced adverse events and were recruited through the Canadian Pharmacogenomics Network for Drug Safety (CPNDS) in multiple Canadian centres in Ontario and British Columbia from 2009–2013 [27].

Patients were included if they had normal kidney function, were treatment-naïve and had received 100 mg/m^2^ cisplatin per cycle. Patients suffering from other diseases than testicular cancer, non-genotyped patients, patients with pre-existing electrolyte disorders or patients that had received abdominal radiation were excluded from this study. All subjects gave their informed consent for inclusion prior their participation in the study. The study was conducted in accordance with the Declaration of Helsinki and the protocol was approved by the UBC C&W Research Ethics Board (ethics certificate no. H04-70358).

### 2.2. Clinical Data Collection

Information concerning co-medication, chemotherapy protocols, duration of the treatment, cumulative dosage of platinum, serum magnesium levels (Mg), serum potassium levels (K), serum sodium levels (Na), serum phosphate levels (PO4) and serum creatinine (SCr) levels was obtained from the medical records. The glomerular filtration rate (GFR) was not available in all patient records. Therefore, estimated glomerular filtration rate (eGFR) was calculated using the CKD-EPI equation [28] as per the KDIGO recommendation [29].

Only cisplatin-induced nephrotoxicity related variables were included as covariates in our analysis (i.e., disease-related variables were excluded). Height and body weight were not included as covariates, as the dosage was already adjusted for this (mg/m^2^). Alcohol consumption, family history of cancer, prior surgery and albumin levels were not available in the medical records and were therefore not be included in the genetic association analyses. All patients in our cohort were cisplatin-naïve at the time of testicular cancer diagnosis.

Age was calculated at the start date of cisplatin treatment. Ethnicity was analysed through ancestry proportions and principal components (PCs) using EIGENSOFT v.5.0 (Harvard and Massachusetts Institute of Technology, Cambridge, MA, USA) and ADMIXTURE. Cardiovascular disease and diabetes data were not available in the database and were for that reason determined based on co-medications [30,31,32,33,34]. Potentially nephrotoxic co-medications were identified from the start until the end date of cisplatin treatment and grouped according to their mechanism of action [35]. The amount of hydration depended on standardized chemotherapy regimen and was derived from Canadian protocols for testicular cancer [36,37]. Cumulative dose was assessed and where carboplatin had been substituted for cisplatin, a conversion factor of 1:4 for cisplatin: carboplatin was used [1,38]. For the baseline SCr and electrolyte measurement, the measurement closest to the start date within 30 days prior to start was taken.

### 2.3. Outcome Definition

To assess the relationship between cisplatin-induced nephrotoxicity and genetic variants, multiple outcomes were studied. Multiple outcomes were used to determine if different cisplatin nephrotoxicity definitions contributed to the variability in effect size and direction of already published associations between these genetic polymorphisms and cisplatin nephrotoxicity. A new tailored definition for cisplatin-induced nephrotoxicity was formulated based on expert opinions to optimize clinical relevance (see below “Adjusted Acute Kidney Injury” Outcome Definition). Since the results for this definition were not comparable with previously published studies, CTCAE-AKI grading and the differences in SCr and eGFR before and after cisplatin-treatment (∆SCr and ∆eGFR) were assessed as well.

#### 2.3.1. “Adjusted Acute Kidney Injury” (Adjusted-AKI) Outcome Definition

The definition of cisplatin-induced nephrotoxicity combines SCr-based staging and electrolyte disturbances (i.e., National Cancer Institute Common Terminology Criteria for Adverse Events (NCI-CTCAE) v.4.03 definitions for electrolyte disturbances) [39] (Table 1). Measurements from start date up to 90 days after the end of cisplatin treatment were collected. The measurement closest to the start date with a cut-off of 30 days was taken as the baseline value when calculating the increase in SCr.

Hyperhydration during administration may cause a hypervolemic state which may provoke hyponatremia [40]. To increase sensitivity and decrease false-positive or overestimated results, hyponatremia must have persisted for longer than two consecutive months. For further statistical analyses, these categories were divided into case, control and ambiguous groups (Table 2). Two separate investigators designated the patients in one of three categories and discrepancies were resolved through discussion between a clinical pharmacologist and nephrologist.

#### 2.3.2. CTCAE-AKI Outcome Definition

The SCr-based CTCAE v.4.03 definition of “Acute Kidney Injury” was also used to define cisplatin-induced nephrotoxicity [39]. Patients were divided into cases (≥grade 1) and controls (<grade 1) (Table 3). The lowest SCr measurement up to 30 days before start of cisplatin treatment was taken as baseline. The follow up value used was the highest SCr value within 90 days after the end of cisplatin treatment.

#### 2.3.3. “∆SCr and ∆eGFR” Outcome Definition

To calculate the differences between baseline and follow up SCr and eGFR (∆SCr and ∆eGFR), the same procedure of creatinine serum measurements was applied as with the CTCAE outcome definition.

### 2.4. Genotype Data

#### 2.4.1. Candidate Genes

The list of candidate genes and related variants was selected from a systematic review [19]. Candidate genes were included if they were found to be significantly associated with nephrotoxicity (any outcome definition) in a published study and the relationship had been replicated at least once. The following five single nucleotide polymorphisms (SNPs) meeting these criteria were included in this study: *ERCC1* rs11615 (chr19:45420395; A>G; a synonymous variant) and rs3212986 (chr19:45409478; C>A/C>G/C>T; non-coding transcript variant), *ERCC2* rs13181 (chr19:45351661; T>A/T>G; stop gained) and rs1799793 (chr19:45364001; C>A/C>T; a missense variant)) and *SLC22A2* rs316019 (chr6:160249250; A>C; a missense variant).

#### 2.4.2. Genotyping

DNA was collected from saliva using Oragene collection kits (DNK Genotek Inc., Ottawa, ON, Canada) and was extracted according to the manufacturer’s protocol. Genomic DNA samples for all patients were genotyped for 7907 variants located within absorption, distribution, metabolism, excretion (ADME) gene regions using the Illumina Infinium Panel (Illumina, San Diego, CA, USA), according to the manufacturer’s instructions at the Canadian Pharmacogenomics Network for Drug Safety at the University of British Columbia. The genotyping details and the ADME custom panel used have been described in previously [27].

#### 2.4.3. Quality Control of Genotype Data

Variants are filtered on SNP call rate (>95%), sample call rate (samples missing ≥2 SNPs excluded), Hardy-Weinberg equilibrium (HWE, *p*-value > 0.05, in controls) and minor allele frequency (MAF, >0.05, in patients with a European proportion ancestry ≥80%). HWE was calculated using Fisher’s exact test.

### 2.5. Statistical Analyses

The genetic association between SNPs and the categorical clinical outcomes reflecting nephrotoxicity (i.e., adjusted-AKI and CTCAE-AKI) were examined using logistic regression assuming an additive model. MAFs for the whole cohort in both designations were calculated for each SNP. An allele frequency lower than 0.5 indicated the minor allele and was also classified as the risk allele. Power analyses were performed assuming a 0.05 significance level, assuming 5% MAF and with an OR > 3 as effect size with the goal of achieving a power of at least 80%. To assess differences between cases and controls (adjusted AKI and CTCAE-AKI designation) for continuous clinical variables, a Mann-Whitney U test was used. The differences between categorical clinical variables and cases and controls (adjusted AKI and CTCAE-AKI designation) were evaluated using a Chi-squared tests. A logistic regression model analysis that included potential confounders (counting subject ancestry) was used to calculate adjusted odds ratios (OR) and the 95% confidence intervals (95% CI) separately for outcomes defined using an adjusted-AKI and CTCAE-AKI designation. Cochran-Armitage trend test was conducted to test the assumption of an additive genetic model. Multiple linear regression was performed to assess the association between genetic variants and the continuous variables ΔeGFR and ΔSCr, adjusting for potential confounders. Key assumptions for multiple linear regression analysis—for example, multivariate normality, no multicollinearity and homoscedasticity—were fulfilled. Clinical variables which caused changes of the crude regression coefficient by 10% or more is considered a confounder and is added to the model. Multiple testing was accounted for using Bonferroni adjustments (*p* = 0.05/5 = 0.01). Statistical analyses were performed using SPSS v.25 (IBM Corporation, Armonk, NY, USA).

## 3. Results

### 3.1. Study Population

The study included 282 testicular-cancer patients from five adult oncology centres in British Columbia and Ontario through active surveillance of the Canadian Pharmacogenomics Network for Drug Safety (CPNDS) [41]. Ambiguous patients (Table 2 and Table 3) or patients with missing SCr or eGFR data were excluded from further analyses. From the primary cohort, 72 patients were excluded because they were not genotyped (N = 61), had received abdominal radiation (N = 4) or because they were not diagnosed with testicular cancer (N = 7). From the secondary cohort (N = 210), 47 patients were excluded for the adjusted-AKI analyses due to pre-existing renal disease, incomplete data regarding the start- and/or end date of cisplatin therapy or absence of laboratory values. For the CTCAE-AKI outcome and ∆SCr analyses, 51 patients were excluded due to lack of SCr data. For the ∆eGFR analyses, 52 patients were excluded due to lack of eGFR data.

**Figure 1:**
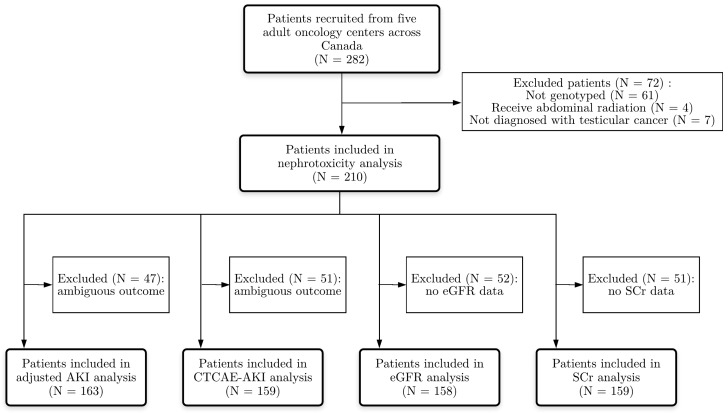
Flowchart of patient inclusion in statistical analyses.

For genetic association analyses 167, 159, 158 and 159, patients were eligible for the adjusted AKI designation, the CTCAE-AKI designation and ∆eGFR and ∆SCr analyses, respectively (Figure 1). These patient cohorts were similar with respect to baseline characteristics. The mean age (± standard deviation) of the testicular-cancer patients was 31.8 ± 10.2 (Table 4). European has the highest ancestry proportion in our dataset (0.72 ± 0.26) followed by South Asian, East Asian, American and African. The detailed of ancestry analysis has been published elsewhere [27]. Patients had a low number of comorbidities: 1.0% (N = 2) suffered from diabetes and 3.3% (N = 7) from a cardiovascular disease. Only 2.9% (N = 6) of the patients received carboplatin within 90 days after cisplatin treatment ended (these dosages were included in the calculation of total platinum exposure). A majority of patients received the regimen of cisplatin with bleomycin and etoposide (BEP; 65%, N = 136). Because data on phosphate levels were missing for 207 patients, this electrolyte was excluded from further analyses.

### 3.2. Genotyping Results

The lowest SNP call rate was 97.5% for *SLC22A2* rs316019 (Table S2). HWE was fulfilled in the control group of all evaluated SNPs for adjusted-AKI outcome (*p* > 0.05) but not in the control group of *ERCC1* rs1799793 for the CTCAE-AKI outcome (*p* = 0.013) (Tables S1 and S2).

### 3.3. Adjusted AKI Analysis

For this outcome, 75 cases and 88 controls were identified (Table S3). Cases had significantly lower baseline magnesium compared to controls (0.83 vs. 0.88 mmol/L, *p* = 0.008). Quinolone usage was significantly higher in cases versus controls (24% vs. 5.7%, *p* = 0.001). Cases received significantly more platinum (400 vs. 300 mg/m^2^, *p* = 0.001) and were treated longer with platinum (4 vs. 3 cycles, *p* = 0.001) compared to controls.

Genetic association analyses on the adjusted AKI designation were corrected for quinolone usage, cumulative dose, baseline magnesium and ancestry using principal components (PC’s) to account for population structure. None of the genetic variants were found to be significantly associated with the risk of nephrotoxicity using this definition (Table 5 and Table 6). In addition, Cochran-Armitage trend test also showed no significant trend to confirm the additive effect of minor allele (Table 6).

### 3.4. CTCAE-AKI Analysis

For this outcome, 36 cases and 123 controls were identified (Table S4). Cases were significantly older compared to controls (35 vs. 29 years old, *p* = 0.002) and differed from controls in ancestry: cases had a lower proportion who were of East-Asian ancestry (0 vs. 0.023, *p* = 0.041) and higher proportion who were of European ancestry (0.853 vs. 0.811, *p* = 0.017). Cases used proton-pump inhibitors (PPIs) significantly more often compared to controls (25% vs. 8%, *p* = 0.015). Cases received significantly more platinum (400 vs. 300 mg/m^2^, *p* = 0.005) and were treated longer with platinum (4 vs. 3 cycles, *p* = 0.007) compared to controls. Furthermore, therapy regimens varied between cases and controls: cases were less often treated with a bleomycin-etoposide-platinum (BEP) protocol (53% vs. 72%, *p* = 0.041) and cases received chemotherapy hydration less often (10.75 (IQR = 10.50–10.75) vs. 10.75 (IQR = 0) L/cycle, *p* = 0.004).

The results of genotypic logistic regression are provided in Table 7. When corrected for age, ancestry from four PCs, chemotherapy protocol, cumulative dosage, hydration and PPI usage, patients carrying *ERCC1* rs3212986 heterozygous genotypes were found to have fewer nephrotoxicity events when compared with patients carrying the homozygous wildtype (OR_adj_ = 0.24, CI = 0.08–0.70, *p* = 0.009). Patients carrying *SLC22A2* rs316019 heterozygous genotypes were found to have a greater number of nephrotoxicity events than patients who carrying the wildtype (normal) genotype before and after adjusting for the same covariates (OR_adj_ = 5.06, CI = 1.69–15.16, *p* = 0.004). Besides this, the *SLC22A2* rs316109 homozygous variant carriers had more nephrotoxicity events than patients carrying the wildtype genotype, however after Bonferroni correction this was no longer statistically significant (OR_adj_ = 38.12, CI = 1.89–767.51, *p* = 0.017).

Additive effect of risk allele was found significant only on *SLC22A2* rs316109. The OR was even higher after adjustment (OR_adj_ = 4.41, CI = 1.96–9.88, *p* < 0.001). In contrast, addition of minor allele on *ERCC1* rs3212986 produce protective effect although the result was not significant (OR_adj_ = 0.52, CI = 0.26–1.07, *p* = 0.076). The additive effect of minor allele was confirmed by Cochran-Armitage trend test but only for *SLC22A2* rs316019 and *ERCC2* rs13181 (Table 8).

### 3.5. ∆SCr and ∆eGFR Analysis

Multiple linear regression was used to predict ∆SCr and ∆eGFR based on genotype for each SNP before and after adjustment for confounding variables. The analysis did not reveal any statistically significant results (Table 9). However, there was a very slight trend for the *ERCC1* rs3212986 variant to be protective and the *SLC22A2* rs316019 homozygous variant to be a risk factor, based on box-plots (Figures S1 and S2).

## 4. Discussion

### 4.1. Main Findings

Previous studies assessing the associations between *ERCC1* rs3212986 and *SLC22A2* rs316019 genotypes and cisplatin-induced nephrotoxicity have reported conflicting results. In this study, associations between genetic variants and multiple definitions of cisplatin-induced nephrotoxicity were analysed in the same dataset and demonstrated that different definitions of cisplatin nephrotoxicity contributed to variability of results. We could not reproduce the same genetic associations that were previously reported, when using the adjusted-AKI or continuous outcomes [13,16,18,42]. In contrast, when using the CTCAE-AKI outcome in the same patient sample, the *ERCC1* rs3212986 heterozygous genotype was reno-protective whilst the *SLC22A2* rs316019 homozygous genotype was a risk factor for cisplatin-induced nephrotoxicity. We also found that additive effect of risk allele was found significant only on *SLC22A2* rs316109.

Several published studies could not detect any significant associations between the CTCAE-AKI outcome definition of cisplatin-induced nephrotoxicity and *ERCC1* rs3212986 [14,43,44,45]; the reasons for this lack of association include lack of study power, population stratification or phenotypic heterogeneity. However, studies carried out by Tzvetkov et al. and Khrunin et al. did reveal associations between *ERCC1* rs3212986 genotypes and cisplatin-induced nephrotoxicity. Tzvetkov et al. found that homozygous variants were not associated with a decrease of eGFR, while the C allele carriers (major allele) had mean decrease of 11.5 ± 1.8% of eGFR (*p* = 0.004) [13]. By applying the same genetic model, we also found that the C allele carriers of this SNP have higher mean eGFR reduction than the homozygous variant subjects although the result was not statistically significant (18.9 ± 22.6 vs. 13.5 ± 23.0 mL/min/1.73 m^2^; *p* = 0.412). This finding suggested protective effect of the variant genotype of rs3212986. Furthermore, we found that variant genotypes were protective against cisplatin nephrotoxicity when applying the CTCAE-AKI definition of nephrotoxicity: heterozygous carriers of the *ERCC1* rs3212986 had an OR_adj_ of 0.24 (95% CI: 0.08–0.70) while the homozygous variant had an OR_adj_ of 0.43 (95% CI: 0.07–2.47; *p* = 0.341). Addition of minor allele on this SNP produce protective effect although the result was not significant (ORadj = 0.52, CI = 0.26–1.07, *p* = 0.076). In contrast to these findings, Khrunin et al. reported a higher prevalence of cisplatin-induced nephrotoxicity among heterozygous genotypes compared with homozygous wildtype (OR = 3.29, 95% CI = 1.40–7.73, *p* = 0.009) [12].

The relationship between *SLC22A2* rs316019 genotypes and cisplatin-induced nephrotoxicity has been assessed in multiple studies. Filipski et al. reported a significant increase in SCr compared to baseline in homozygous wildtype patients after the first cycle (*p* = 0.0009) but found no significant increase in heterozygous patients (*p* = 0.12) [18]. Iwata et al. reported a significant higher increase in SCr in homozygous wildtype patients compared to heterozygous patients (0.34 ± 0.33 vs. 0.14 ± 0.12 mg/dL, *p* = 0.04, respectively) [16]. In addition, Zhang et al. observed a higher increase of cystatin C in homozygous wildtype patients compared to heterozygous and homozygous variant patients (0.043 ± 0.107 vs. −0.013 ± 0.120 mmol/L, *p* = 0.009, respectively) [46]. These results indicate that the homozygous wildtype genotype may be a risk factor for developing cisplatin-induced nephrotoxicity. In contrast, our results suggest that both homozygous and heterozygous variant carriers have an increased risk of cisplatin-induced nephrotoxicity when using the CTCAE-AKI definition. This finding also supported by significant additive effect of risk allele on *SLC22A2* rs316109 when applying additive genetic model. However, our study identified a possible greater risk of nephrotoxicity as defined by ∆SCr in patients carrying the homozygous variant (Figure S1); these data are consistent with Zhang et al. and Hinai et al., who reported a higher increase of SCr in heterozygous and homozygous variant than in homozygous wildtype subjects, although the result is not statistically significant (0.83 ± 7.39 vs. 2.09 ± 6.30 mmol/L, *p* = 0.35 and 0.30 ± 0.30 vs. 0.40 ± 0.53 mg/dL, *p* = 0.25, respectively) [46].

Other factors could also contribute to the discrepancy in results between our study and previous studies. Our results suggest that cisplatin-induced nephrotoxicity is confounded by ethnic origin. The CTCAE-AKI outcome was related to East-Asian and European ancestry. Our results suggest that East-Asian ancestry may be a protective factor and European ancestry may be a risk factor for cisplatin-induced nephrotoxicity. This may also explain the differences of results between our study and the studies of Iwata et al. and Hinai et al. that included subjects of East Asian ancestry. Discrepancies regarding the ΔSCr—and hence, ΔeGFR—among those studies could also be explained by the age of the population. Hinai et al. and Iwata et al. both studied an older population: 68.0 ± 9.7 and 65.8 ± 7.7 years old (mean ± SD) [16,42]. As highlighted before, older age could attribute to a higher increase in SCr [47]. This may explain the elevated SCr levels in the wildtype homozygous group of *SLC22A2* rs316019 found by Iwata et al. and Hinai et al. compared to our study. Furthermore, our population received a high dose cisplatin (100 mg/m^2^ per cycle) compared to dosages used in other indications and compared to the other studies [16,42]: patients analysed by Hinai et al. received 80 mg/m^2^ per cycle and Iwata et al. treated their patients with 60–80 mg/m^2^ cisplatin per cycle. The higher cisplatin-dose in our study could have attributed to a possible higher incidence of cisplatin-induced nephrotoxicity.

This study further shows that different outcome definitions produce different results. The main difference in the outcomes definitions is the inclusion of electrolyte disturbances in the adjusted AKI outcome definition (Table 10). Our results suggest that the genetic associations were found when the SCr based definition was used but not when using an electrolyte-based definition that forms the adjusted AKI outcome definition. Acute kidney injury caused by cisplatin mainly manifests itself as renal tubular injury and is therefore characterized earlier by electrolyte abnormalities (phosphate, magnesium, potassium and sodium) [48]. However, incorporating serum abnormalities with creatinine serum levels in one single definition of cisplatin nephrotoxicity should be further validated.

### 4.2. Gene Expression and Regulation

*ERCC1* rs3212986, located at the 3′ UTR (non-coding region) was not associated with changes in protein and mRNA expression [49,50]. However, the tissue expression quantitative trait loci (eQTL) analysis from the Genotype-Tissue Expression (GTEx) Project reported a significant association between rs3212986 and gene expression in various tissues [51]. Unfortunately, no association has been found between rs3212986 and ERCC1 expression in kidney cortex tissue. *SLC22A2* rs316019, a nonsynonymous missense mutation (p.270Ala>Ser), is the only common coding polymorphism of *SLCC2A2* with an allele frequency ranging from 9–16% and is reported to cause changes in transporter function [52]. No significant eQTLs were found for rs316019 in the eQTL tissues database [51]. Specific functional validation of *ERCC1* rs3212986 and *SLC22A2* rs316019 in kidney tubular tissue is needed to elucidate their role in cisplatin nephrotoxicity and how they affect protein expression involved in cisplatin nephrotoxicity pathway (e.g., OCT2).

### 4.3. Strength and Limitations of the Study

Compared to the previously published studies, our study was conducted in an appropriate population of relatively young adult male patients, who had a low number of comorbidities. By studying a dataset of testicular cancer patients, we minimized the influence of gender, older age, comorbidities and long-term use of medications that could have affected the renin-angiotensin systems (e.g., angiotensin converting enzyme inhibitors and angiotensin receptor blockers) and nephrotoxic compounds (e.g., non-steroidal anti-inflammatory drugs).

Our study had several limitations. The retrospective design led to several potential but unavoidable bias. Since laboratory measurements that were available were mostly measured in patients who were monitored more intensively, any missing data was non-random. Hence, the measurements that were available more likely to found individuals who were prone to cisplatin-induced nephrotoxicity. This resulted in selection bias and a possible overestimation of the amount of cases. The relatively small sample size was also a possible cause of failing to detect an association in this candidate gene study. A large prospective cohort study with a genome-wide approach is recommended to explore additional genetic variants that might be of importance. Furthermore, slightly different number of cohorts were used for each outcome. This may also have influenced the associations observed with each outcome.

## 5. Conclusions

In conclusion, the results of this study imply that the use of different outcome definitions lead to altered results. Consensus on a set of clinically relevant outcome definitions that future studies can follow are needed. The adjusted acute kidney injury definition that includes electrolyte imbalances seems more appropriate for cisplatin-induced nephrotoxicity. However, further validation of the definition and staging is necessary before it can be applied in further research or clinical settings. Furthermore, this study provides more evidence for associations between genetic variants and cisplatin-induced nephrotoxicity by using serum creatinine-based grading. These findings imply that genetic variations are involved in the inter-individual susceptibility to cisplatin-induced nephrotoxicity. Thus, in the future genotyping will make it possible to optimize therapy with cisplatin for the individual patients by improving cisplatin dosage selection–lower doses for patients prone to renal toxicity and higher doses for patient not susceptible to developing renal toxicity.

## Figures and Tables

**Table 1:** Adjusted Acute Kidney Injury (Adjusted-AKI) grading.

Grade	Definition	Characteristic(s)
0	An increase in serum creatinine, up to 1.5 times baseline value AND Electrolyte disorders grade 0 CTCAE: • Hypomagnesemia: ≥LLN–1.2 mg/dL; <LLN–0.5 mmol/L, OR • Hypokalaemia: ≥LLN–3.0 mmol/L, OR • Hypophosphatemia: ≥LLN–2.5 mg/dL; <LLN–0.8 mmol/L, OR • Hyponatremia: ≥LLN–130 mmol/L (>2 months)	Asymptomatic
1	Between 1.5–1.9 times baseline SCr OR ≥0.3 mg/dL (≥26.5 µmol/L) increase in SCr OR Electrolyte disorders grade 1 CTCAE: • Hypomagnesemia: <LLN–1.2 mg/dL; <LLN–0.5 mmol/L, OR • Hypokalaemia: <LLN–3.0 mmol/L, OR • Hypophosphatemia: <LLN–2.5 mg/dL; <LLN–0.8 mmol/L, OR • Hyponatremia: <LLN–130 mmol/L (>2 months)	Possible Symptomatic
2	An increase in serum creatinine between 2.0–2.9 times baseline SCr ORElectrolyte disorders grade 2 CTCAE: • Hypomagnesemia: <1.2–0.9 mg/dL; <0.5–0.4 mmol/L, OR • Hypokalaemia: <LLN–3.0 mmol/L, OR • Hypophosphatemia: <2.5–2.0 mg/dL; <0.8–0.6 mmol/L, OR • Hyponatremia: <LLN–130–120 mmol/L (>2 months)	Clinically relevant, required intervention
3	An increase in serum creatinine at least 3.0 times baseline OR Increase in serum creatinine to ≥4.0 mg/dL (≥353.6 µmol/L) OR Initiation of renal replacement therapy, OR OR Electrolyte disorders ≥grade 3 CTCAE: • Hypomagnesemia: <0.9 mg/dL; <0.4 mmol/L, OR • Hypokalaemia: <3.0 mmol/L; hospitalization indicated, OR • Hypophosphatemia: <2.0 mg/dL; <0.6 mmol/L, OR • Hyponatremia: <LLN–120 mmol/L (>2 months)	Required close monitoring

**Table 2:** Case-control designation according to Adjusted-AKI outcome definition.

Case	Control	Ambiguous
Acute nephrotoxicity ≥ grade 1 OR Received electrolyte supplementation	Acute nephrotoxicity < grade 1 AND No supplementation	No lab values available during the time frame (3 months before initiation and 3 months after the last administration of cisplatin) OR Incomplete data e.g., initiation and end date of cisplatin therapy OR Pre-existing renal disease (electrolyte disturbances, not SCr or eGFR)

SCr: serum creatinine; eGFR: estimated glomerular filtration rate.

**Table 3:** Case-control designation according to Common Terminology Criteria for Adverse Events (CTCAE)-AKI Outcome Definition.

Case	Control	Ambiguous
Acute kidney injury ≥ grade 1	Acute kidney injury < grade 1	No lab values available during the time frame (3 months before initiation and 3 months after the last administration of cisplatin) OR Incomplete data e.g., initiation and end date of cisplatin therapy

**Table 4:** Clinical characteristics testicular cancer patients included in nephrotoxicity analyses (N = 210).

Characteristics		
Age at start treatment, mean ± SD, years		31.8 ± 10.2
Ancestry, mean ± SD, proportion	European	0.72 ± 0.26
East-Asian		0.09 ± 0.23
American		0.05 ± 0.10
African		0.03 ± 0.03
South-Asian		0.11 ± 0.15
Cardiovascular disease, no. (%)		7 (3.3)
Diabetes, no. (%)		2 (1.0)
Potentially nephrotoxic co-medications, mean ± SD, total number per patient		2 ± 2
Potentially nephrotoxic co-medications, no. (%)	ACEIs ^a^	3 (1.4)
Aminoglycosides		4 (1.9)
ARBs ^b^		1 (0.5)
Benzodiazepines		30 (14)
NSAIDs ^c^		6 (2.9)
Betalactams		26 (12)
PPIs ^d^		25 (12)
Quinolones		29 (14)
Statins		2 (1.0)
Acetaminophen		29 (14)
Other		104 (50)
Baseline [SCr], mean ± SD, umol/L		84 ± 16
Baseline [K^+^], mean ± SD, mmol/L		4.1 ± 0.4
Baseline [Mg^2+^], mean ± SD, mmol/L		0.85 ± 0.10
Baseline [Na^+^], mean ± SD, mmol/L		138 ± 2.49
Baseline [PO4^-^], mean ± SD, mmol/L		1.09 ± 0.23
Cumulative platinum dose, mean ± SD, mg/m^2^		380 ± 123
Duration cisplatin treatment, mean ± SD	Weeks	8.7 ± 3.3
Cycles		3.8 ± 1.1
Chemotherapy protocol, no. (%), BEP		136 (65)
Chemotherapy hydration, mean ± SD, L/cycle		10.7 ± 0.5

^a^ ACEIs: Angiotensin-converting enzyme inhibitors, ^b^ ARBs: Angiotensin-II-Receptor Blockers, ^c^ NSAIDs: non-steroidal anti-inflammatory drugs, ^d^ PPIs: proton-pump inhibitors, BEP: bleomycin, etoposide, and cisplatin.

**Table 5:** Strength of genotypic association between genetic polymorphisms and cisplatin nephrotoxicity in adjusted-AKI outcome (N = 163).

Gene–SNP	OR	95% CI	*p*-Value	OR_adj_	95% CI_adj_	*p*-Value_adj_
*ERCC1* rs11615						
GG	1 ^#^			1 ^#^		
GA	1.30	0.63–2.67	0.48	1.45	0.64–3.27	0.38
AA	1.24	0.51–3.02	0.63	1.47	0.50–4.28	0.48
*ERCC1* rs3212986						
CC	1 ^#^			1 ^#^		
CA	0.71	0.37–1.36	0.31	0.63	0.30–1.34	0.23
AA	1.00	0.30–3.37	1.00	1.44	0.32–6.43	0.63
*ERCC2* rs13181						
AA	1 ^#^			1 ^#^		
CA	0.84	0.42–1.66	0.61	0.59	0.26–1.33	0.20
CC	1.60	0.65–3.93	0.31	1.43	0.50–4.07	0.51
*ERCC2* rs1799793						
AA	1 ^#^			1 ^#^		
CA	1.00	0.49–2.03	1.00	0.92	0.40–2.15	0.85
CC	0.50	0.21–1.17	0.11	0.55	0.21–1.43	0.22
*SLC22A2* rs316019						
CC	1 ^#^			1 ^#^		
AC	1.15	0.51–2.57	0.71	1.10	0.43–2.79	0.85
AA	2.46	0.22–27.78	0.47	1.70	0.11–25.57	0.70

_adj_ Adjusted for: cumulative dose, quinolone usage, all ancestries (from four PCs) and baseline magnesium. ^#^ Reference category.

**Table 6:** Odds ratio of minor allele addition in adjusted-AKI outcome (N = 163) and Cohcran-Armitage trend test result for additive model assumption.

Gene–SNP	OR	95% CI	*p*-Value	OR_adj_	95% CI_adj_	*p*-Value_adj_	Cohcran-Armitage Trend Test *p*-Value
*ERCC1* rs11615 GG vs. GA vs. AA	1.13	0.73–1.75	0.586	1.23	0.73–2.05	0.436	0.586
*ERCC1* rs3212986 AA vs. CA vs. CC	0.86	0.54–1.40	0.551	0.89	0.51–1.54	0.669	0.537
*ERCC2* rs13181 CC vs. CA vs. AA	1.19	0.75–1.88	0.461	1.04	0.61–1.78	0.875	0.497
*ERCC2* rs1799793 CC vs. CA vs. AA	0.70	0.45–1.09	0.114	0.73	0.44–1.19	0.206	0.280
*SLC22A2* rs316019 AA vs. CA vs. CC	1.28	0.64–2.59	0.488	1.17	0.53–2.60	0.702	0.502

_adj_ Adjusted for: cumulative dose, quinolone usage, all ancestries (as PC’s) and baseline magnesium.

**Table 7:** Strength of genotypic association between genetic polymorphisms and cisplatin nephrotoxicity in CTCAE-AKI designation (N = 159).

Gene–SNP	OR	95% CI	*p*-Value	OR_adj_	95% CI_adj_	*p*-Value_adj_
*ERCC1* rs11615						
GG	1 ^#^			1 ^#^		
GA	1.30	0.57–2.99	0.55	1.23	0.45–3.39	0.68
AA	0.48	0.14–1.65	0.24	0.53	0.12–2.37	0.41
*ERCC1* rs3212986						
CC	1 ^#^			1 ^#^		
CA	0.45	0.20–1.02	0.06	0.24	0.08–0.70	0.009 *
AA	0.48	0.10–2.36	0.37	0.43	0.07–2.47	0.34
*ERCC2* rs13181						
AA	1 ^#^			1 ^#^		
CA	1.16	0.49–2.73	0.74	0.59	0.20–1.76	0.37
CC	3.16	1.17–8.58	0.02	1.72	0.53–5.65	0.35
*ERCC2* rs1799793						
AA	1 ^#^			1 ^#^		
CA	1.52	0.65–3.54	0.33	2.39	0.84–6.77	0.10
CC	0.57	0.18–1.79	0.33	0.66	0.16–2.64	0.56
*SLC22A2* rs316019						
CC	1 ^#^			1 ^#^		
AC	3.24	1.36–7.74	0.008 *	5.06	1.69–15.16	0.004 *
AA	9.18	0.80–105.80	0.08	38.12	1.89–767.51	0.02

_adj_ Adjusted for: age, all ancestries (as PC’s), chemotherapy protocol, cumulative dosage, hydration and PPI usage, ^#^ Reference category, * significant (*p* < 0.01).

**Table 8:** Odds ratio of minor allele addition in CTCAE-AKI designation (N = 159) and Cohcran-Armitage trend test result for additive model assumption.

Gene–SNP	OR	95% CI	*p*-Value	OR_adj_	95% CI_adj_	*p*-Value_adj_	Cohcran-Armitage Trend Test *p*-Value
*ERCC1* rs11615 GG vs. GA vs. AA	0.78	0.46–1.33	0.364	0.92	0.50–1.68	0.777	0.368
*ERCC1* rs3212986 AA vs. CA vs. CC	0.57	0.30–1.06	0.077	0.52	0.26–1.07	0.076	0.067
*ERCC2* rs13181 CC vs. CA vs. AA	1.84	1.07–3.15	0.027	1.39	0.75–2.58	0.293	0.039 *
*ERCC2* rs1799793 CC vs. CA vs. AA	0.81	0.48–1.38	0.447	0.85	0.47–1.53	0.578	0.473
*SLC22A2* rs316019 AA vs. CA vs. CC	3.29	1.60–6.81	0.001 **	4.41	1.96–9.88	<0.001 **	0.001 **

_adj_ Adjusted for: age, all ancestries (as PC’s), chemotherapy protocol, cumulative dosage, hydration and PPI usage, * significant (*p* < 0.05); proof of trend, ** significant (*p* < 0.01).

**Table 9:** Multiple linear regression analysis results between genetic polymorphisms and ∆SCr and ∆eGFR.

Gene–SNP	∆SCr ^a^				∆eGFR ^b^			
	R^2^	*p*-Value	R^2^_adj_	*p*-Value _adj_	R^2^	*p*-Value	R^2^_adj_	*p*-Value _adj_
*ERCC1* rs11615 GG vs. GA vs. AA	0.01	0.218	0.055	0.17	0.006	0.347	0.042	0.20
*ERCC1* rs3212986 AA vs. CA vs. CC	0.008	0.268	0.058	0.16	0.013	0.167	0.052	0.12
*ERCC2* rs13181 CC vs. CA vs. AA	0.001	0.652	0.046	0.28	0	0.796	0.035	0.29
*ERCC2* rs1799793 CC vs. CA vs. AA	0.001	0.77	0.046	0.27	0.001	0.668	0.036	0.29
*SLC22A2* rs316019 AA vs. CA vs. CC	0.002	0.599	0.047	0.27	0.006	0.343	0.039	0.25

^a^ adjusted for cardiovascular disease, duration (weeks), aminoglycoside users and baseline magnesium, ^b^ adjusted for duration (weeks), baseline potassium and beta-lactams use.

**Table 10:** Multiple outcome definitions of cisplatin-induced nephrotoxicity used in this study.

	Adjusted-AKI	CTCAE-AKI	ΔeGFR	ΔSCr
**Basis of Determination**	SCr + Mg/K/PO_4_/Na	SCr	CKD-EPI equation (SCr+age+sex+ethnicity)	SCr
**Data Characteristics**	Categorical	Categorical	Continuous	Continuous
**Advantage**	Tailored on cisplatin-induced nephrotoxicity	• Mostly used in clinics and studies in cancer subjects • Easily calculated	• Easily calculated • CKD-EPI is the equation recommended by KDIGO	• Routinely measured in patients
**Disadvantage**	• Not comparable with other studies • Not validated yet	• Is ≥ grade 1 cut-off clinically relevant? • SCr often increase late resulting in failing to detect early stage nephrotoxicity	• Could not correct for cystatin-C due to unavailable data in routine practice • Disregarding the clinical value of baseline eGFR	• Highly influenced by various individual factors (e.g., age, gender, body weight, diet etc.) • SCr often increase late resulting in failing to detect early stage nephrotoxicity • Disregarding the clinical value of baseline SCr

## Supplementary Materials

The following are available online at https://www.mdpi.com/2073-4425/10/5/364/s1, Table S1: Genotyping details for adjusted-AKI outcome in patients with European proportion ancestry ≥80% (N = 93), Table S2: Genotyping details for AKI-CTCAE outcome in patients with European proportion ancestry ≥80% (N = 88), Table S3: Clinical characteristics of cases and controls in adjusted-AKI outcome (N = 163), Table S4: Clinical characteristics of cases and controls in AKI-CTCAE outcome (N = 159), Figure S1: Boxplot chart of SCr elevation (∆SCr) by genotype of studied SNPs, Figure S2: Boxplot chart of eGFR reduction (∆eGFR) by genotype of studied SNPs.
