# Supplementary material for: Outcome Definition Influences the Relationship between Genetic Polymorphisms of ERCC1, ERCC2, SLC22A2 and Cisplatin Nephrotoxicity in Adult Testicular Cancer Patients
Source: Genes (Basel). 2019 May 10;10(5):364. doi: 10.3390/genes10050364 (PMC6562793; doi:10.3390/genes10050364)
Supplement: Supplementary file 1 [file genes-10-00364-s001.pdf]

genes

Supplementary Materials:

Outcome Definition Influences the Relationship Between Genetic Polymorphisms of *ERCC1*, *ERCC2*, *SLC22A2* and Cisplatin Nephrotoxicity in Adult Testicular Cancer Patients

Zulfan Zazuli, Leila S. Otten, Britt I. Drogemoller, Mara Medeiros, Jose G. Monzon, Galen E. B. Wright, Christian K. Kollmannsberger, Philippe L. Bedard, Zhuo Chen, Karen A. Gelmon, Nicole McGoldrick, Abhijat Kitchlu, Susanne J.H. Vijverberg, Rosalinde Masereeuw, Colin J.D. Ross, Geoffrey Liu, Bruce C. Carleton, and Anke H. Maitland-van der Zee

Table S1. Genotyping details for adjusted-AKI outcome in patients with European proportion ancestry ≥80% (N=93).

Gene–rsID	SNP Call Rate (%)	Minor Allele Frequency Observed		Minor Allele Frequency Observed (Expected)	HWE *p*-value		Minor Allele Frequency Observed (Expected)	HWE *p*-value
	In Total Cohort (N = 93)		In Controls (N = 46)			In Cases (N = 47)		
ERCC1 rs11615	100	0.392	G	0.337 (0.337)	0.883	G	0.447 (0.447)	0.414
ERCC1 rs3212986	99.5	0.285	A	0.239 (0.238)	0.609	A	0.330 (0.330)	0.558
ERCC2 rs13181	99.5	0.386	C	0.378 (0.378)	0.317	C	0.394 (0.394)	0.023*
ERCC2 rs1799793	100	0.457	C	0.489 (0.424)	0.077	C	0.423 (0.426)	0.137
SLC22A2 rs316019	98.1	0.110	A	0.057 (0.056)	0.690	A	0.160 (0.160)	0.049*

* significant ($p < 0.05$), a-AKI: adjusted acute kidney-injury, SNP: single nucleotide polymorphism, HWE: Hardy-Weinberg equilibrium.

Table S2. Genotyping details for AKI-CTCAE outcome in patients with European proportion ancestry ≥80% (N = 88).

Gene–rsID	SNP Call Rate (%)	Minor Allele Frequency Observed		Minor Allele Frequency Observed (Expected)	HWE *p*-value		Minor Allele Frequency Observed (Expected)	HWE *p*-value
	In Total Cohort (N = 93)		In Controls (N = 46)			In Cases (N = 47)		
ERCC1 rs11615	100	0.369	G	0.359 (0.360)	0.690	G	0.396 (0.398)	0.133
ERCC1 rs3212986	100	0.267	A	0.273 (0.273)	0.445	A	0.250 (0.250)	0.586
ERCC2 rs13181	99.4	0.356	C	0.325 (0.326)	0.181	C	0.438 (0.438)	0.046*
ERCC2 rs1799793	100	0.466	C	0.484 (0.484)	0.013*	C	0.417 (0.419)	0.889
SLC22A2 rs316019	97.5	0.099	A	0.040 (0.040)	0.740	A	0.250 (0.250)	0.586

* significant ($p < 0.05$), a-AKI: adjusted acute kidney-injury, SNP: single nucleotide polymorphism, HWE: Hardy-Weinberg equilibrium.

Table S3. Clinical characteristics of cases and controls in adjusted-AKI outcome (N = 163).

Characteristics		Cases (N = 75)[a]	Controls (N = 88)[a]	*p*-value[b]
Age at start treatment, median (IQR), years		31 (24–40)	30 (24–37)	0.091
Ancestry, median (IQR), proportion	European	0.84 (0.75–0.88)	0.81 (0.65–0.87)	0.267
East-Asian		0.01 (0–0.05)	0.02 (0–0.08)	0.348
American		0.02 (0–0.05)	0.02 (0–0.04)	0.581
African		0.03 (0.01–0.05)	0.03 (0.01–0.05)	0.819
South-Asian		0.08 (0.03–0.14)	0.07 (0.02–0.13)	0.641
Cardiovascular disease, no. (%), yes (vs. no)		3 (4.0)	2 (2.3)	0.662
Diabetes, no. (%), yes (vs. no)		2 (2.7)	0 (0)	0.210
Concomitant nephrotoxic medication, no. (%), yes (vs. no)	ACEIs	2 (2.7)	0 (0)	0.210
Aminoglycosides		3 (4.0)	1 (1.1)	0.335
ARBs		1 (1.3)	0 (0)	0.460
Benzodiazepines		8 (10.7)	11 (12.5)	0.809
NSAIDs		3 (4.0)	3 (3.4)	1.000
Betalactams		9 (12.0)	11 (12.5)	1.000
PPIs		11 (14.7)	8 (9.1)	0.330
Quinolones		18 (24.0)	5 (5.7)	0.001*
Statins		1 (1.3)	1 (1.1)	1.000
Acetaminophen		13 (17.3)	6 (6.8)	0.050
Other		44 (58.7)	44 (50)	0.275
Baseline [SCr], median (IQR), umol/L		83.5 (72–92)	82.5 (74-90)	0.713
Baseline [K], median (IQR), mmol/L		4.1 (3.9–4.4)	4.1 (3.9–4.3)	0.707
Baseline [Mg], median (IQR), mmol/L		0.83 (0.77–0.90)	0.88 (0.82–0.94)	0.008*
Baseline [Na+], median (IQR), mmol/L		139 (137–140)	138 (137–141)	0.480
Cumulative platinum dose, median (IQR), mg/m^2		400 (300–400)	300 (300–400)	0.001*
Duration cisplatin treatment, median (IQR)	weeks	9.6 (6.6–9.75)	6.7 (6.6–9.6)	0.151
cycles		4 (3–4)	3 (3–4)	0.001*
Chemotherapy protocol, no. (%), BEP (vs. other)		46 (61.3)	65 (73.9)	0.095
Chemotherapy hydration, median (IQR), L/cycle		10.75 (10.70–10.75)	10.75 (10.75–10.75)	0.129
ERCC1 rs11615, no. (%)	GG	16 (21.33)	18 (20.45)	
GA		39 (52)	42 (47.73)	
AA		20 (26.67)	28 (31.82)	
ERCC1 rs3212986 (N=1 failed genotyping), no. (%)	AA	6 (8)	6 (6.90)	
AC		30 (40)	42 (48.28)	
CC		39 (52)	39 (44.83)	
ERCC2 rs13181 (N=1 failed genotyping), no. (%)	CC	15 (20)	11 (12.64)	
CA		25 (33.33)	35 (40.23)	
AA		35 (46.67)	41 (47.13)	
ERCC2 rs1799793, no. (%)	CC	13 (17.33)	26 (29.55)	
CA		35 (46.67)	35 (39.77)	
AA		27 (36)	27 (30.68)	
SLC22A2 rs316019 (N=4 failed genotyping), no. (%)	AA	2 (2.74)	1 (1.16)	
AC		14 (19.18)	15 (17.44)	
CC		57 (78.08)	70 (81.40)	

* significant, IQR: Interquartile Range, [a] Percentages given are percentages within cases and controls, [b] Categorical variables were analyzed using a 2-sided Fisher's exact test. Continuous variables were analyzed using Mann-Whitney U Test.

Table S4. Clinical characteristics of cases and controls in AKI-CTCAE outcome (N = 159).

Characteristics		Cases (N = 36)[a] CTCAE grade ≥ 1	Controls (N = 123)[a] CTCAE grade 0	*p*-value[b]
Age at start treatment, median (IQR), years		35 (28–46)	29 (23–37)	0.002*
Ancestry, median (IQR), proportion	European	0.853 (0.768–0.889)	0.811 (0.637–0.862)	0.017*
East-Asian		0 (0–0.034)	0.023 (0.072)	0.041*
American		0.024 (0–0.054)	0.022 (0-0.048)	0.878
African		0.033 (0.008–0.053)	0.031 (0.01–0.048)	0.895
South-Asian		0.06 (0.024–0.095)	0.077 (0.025–0.139)	0.415
Cardiovascular disease, no. (%), yes (vs. no)		3 (8.3)	2 (1.6)	0.077
Diabetes, no. (%), yes (vs. no)		1 (2.8)	1 (0.8)	0.403
Concomitant nephrotoxic medication, no. (%), yes (vs. no)				
ACEIs		2 (5.6)	0 (0)	0. 05

Aminoglycosides		1 (2.8)	3 (2.4)	1.000
ARBs		1 (2.8)	0 (0)	0.226
Benzodiazepines		6 (16.7)	13 (10.6)	0.380
NSAIDs		2 (5.6)	4 (3.3)	0.619
Betalactams		4 (11.1)	14 (11.4)	1.000
PPIs		9 (25)	10 (8.1)	0.015*
Quinolones		9 (25)	13 (10.6)	0.051
Statins		1 (2.8)	1 (0.8)	0.403
Acetaminophen		9 (25)	13 (10.6)	0.051
Other		23 (63.9)	63 (51.2)	0.19
Baseline [SCr], median (IQR), umol/L		82 (72–95)	83 (74–90)	0.789
Baseline [K], median (IQR), mmol/L		4.1 (3.9–4.3)	4.1 (3.9–4.3)	0.970
Baseline [Mg], median (IQR), mmol/L		0.81 (0.75–0.9)	0.86 (0.8–0.93)	0.054
Baseline [Na+], median (IQR), mmol/L		139 (136–141)	139 (137–140)	0.792
Cumulative platinum dose, median (IQR), mg/m^2		400 (300–437)	300 (300–400)	0.005*
Duration cisplatin treatment, median (IQR)	weeks	10 (7–10)	10 (7–10)	0.639
	cycles	4 (3–4)	3 (3–4)	0.007*
Chemotherapy protocol, no. (%), BEP (vs. other)		19 (52.8)	89 (72.4)	0.041*
Chemotherapy hydration, median (IQR), L/cycle		10.75 (10.5–10.75)	10.75 (0)	0.004*
ERCC1 rs11615, no. (%)	AA	11 (30.6)	38 (30.9)	
	GA	21 (58.3)	56 (45.5)	
	GG	4 (11.1)	29 (23.6)	
ERCC1 rs3212986, no. (%)	CC	23 (63.9)	55 (44.7)	
	CA	11 (30.6)	58 (47.2)	
	AA	2 (5.6)	10 (8.1)	
ERCC2 rs13181, no. (%)	AA	14 (38.9)	62 (50.8)	
	CA	12 (33.3)	46 (37.7)	
	CC	10 (27.8)	14 (11.5)	
ERCC2 rs171140, no. (%)	AA	11 (30.6)	41 (33.3)	
	CA	20 (55.6)	49 (39.8)	
	CC	5 (13.9)	33 (26.8)	
SLC22A2 rs316019, no. (%)	CC	22 (61.1)	101 (84.9)	
	CA	12 (33.3)	17 (14.3)	
	AA	2 (5.6)	1 (0.8)	

* significant, IQR: Interquartile Range, [a] Percentages given are percentages within cases and controls, [b] Categorical variables were analyzed using a 2-sided Fisher's exact test. Continuous variables were analyzed using Mann-Whitney U Test.

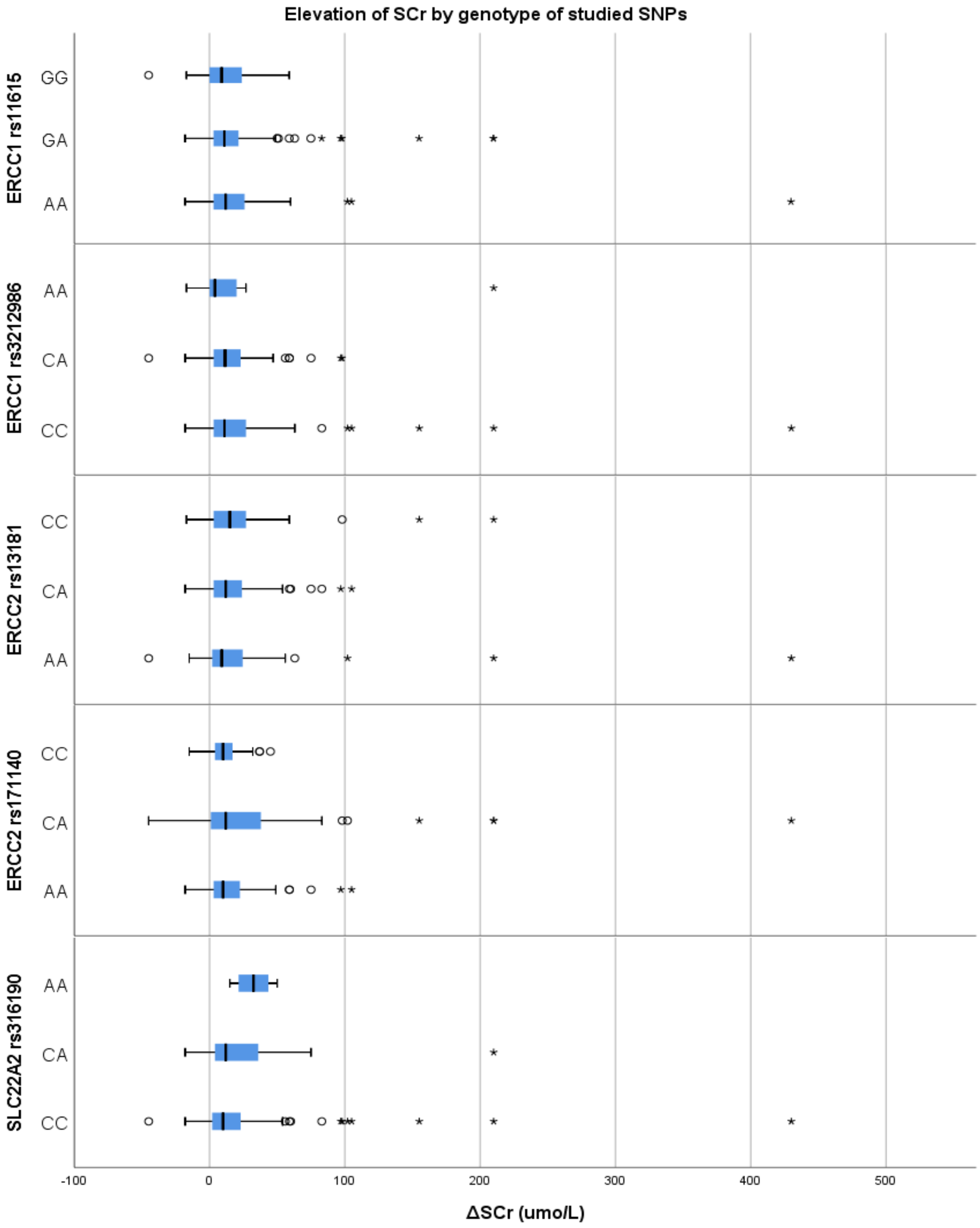

Figure S1. Boxplot chart of SCr elevation (ΔSCr) by genotype of studied SNPs.

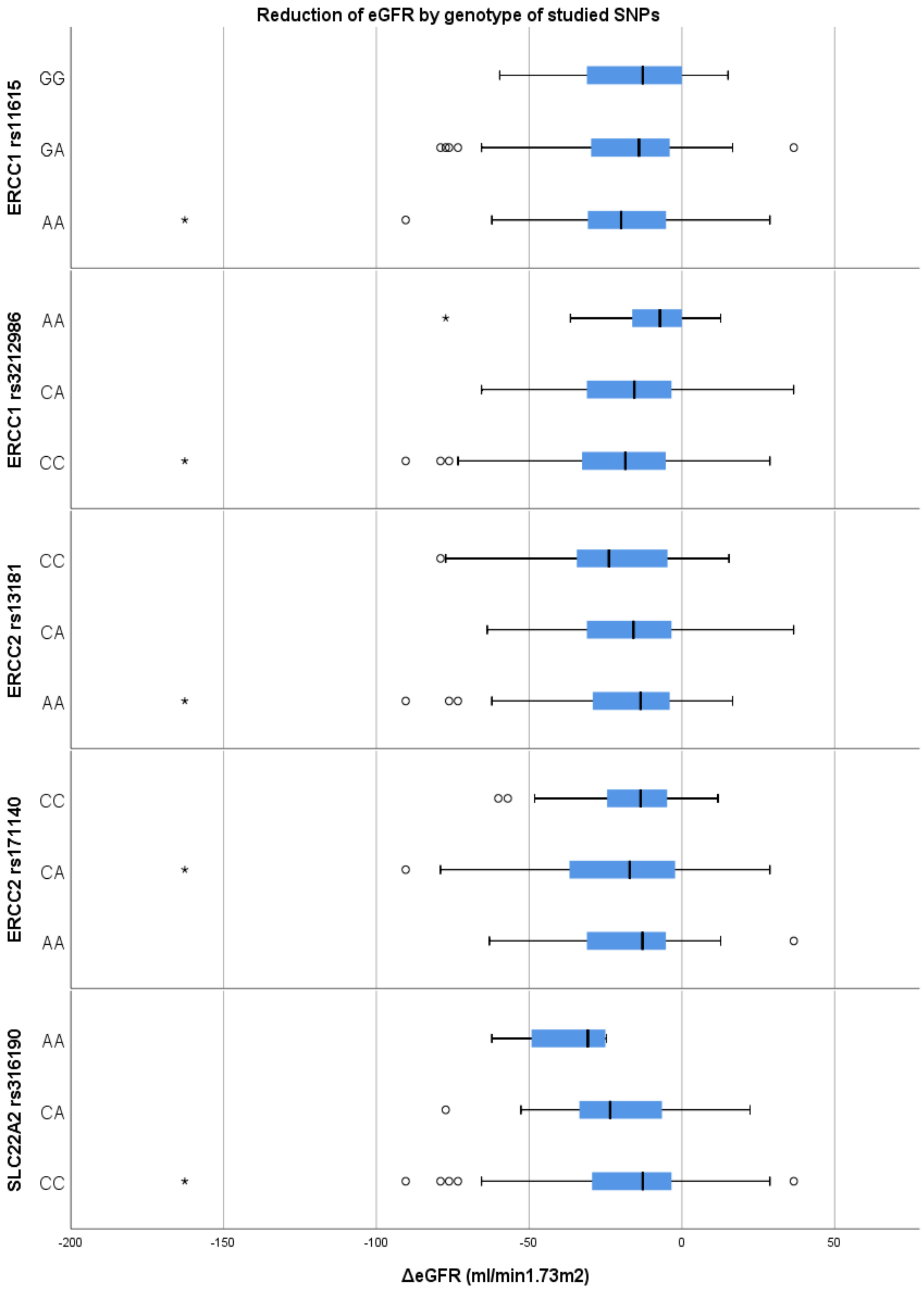

Figure S2. Boxplot chart of eGFR reduction (ΔeGFR) by genotype of studied SNPs.
